# High Concentration of Anti-SARS-CoV-2 Antibodies 2 Years after COVID-19 Vaccination Stems Not Only from Boosters but Also from Widespread, Often Unrecognized, Contact with the Virus

**DOI:** 10.3390/vaccines12050471

**Published:** 2024-04-28

**Authors:** Jakub Swadźba, Andrzej Panek, Paweł Wąsowicz, Tomasz Anyszek, Emilia Martin

**Affiliations:** 1 Medical Faculty, Andrzej Frycz Modrzewski Krakow University, 30-705 Krakow, Poland; jakub.swadzba@diag.pl (J.S.); tomasz.anyszek@diag.pl (T.A.); 2 Medical Department Diagnostyka S.A., 31-864 Krakow, Poland; andrzej.panek@diag.pl (A.P.); pawel.wasowicz@diag.pl (P.W.)

**Keywords:** SARS-CoV-2 IgG, COVID-19 vaccination, spike antibody, nucleocapsid antibody, booster dose, humoral immunity, Comirnaty, breakthrough infections

## Abstract

This study follows 99 subjects vaccinated with Pfizer/BioNTech COVID-19 vaccines over two years, with particular focus on the last year of observation (between days 360 and 720). The response to the vaccination was assessed with Diasorin’s SARS-CoV-2 TrimericSpike IgG. Screening for SARS-CoV-2 infection was performed with Abbott’s SARS-CoV-2 Nucleocapsid IgG immunoassay. Data from questionnaires were also analyzed. Two years after the first vaccine dose administration, 100% of the subjects were positive for anti-spike SARS-CoV-2 IgG and the median antibody level was still high (3600 BAU/mL), dropping insignificantly over the last year. Simultaneously, a substantial increase in seropositivity in anti-nucleocapsid SARS-CoV-2 IgG was noted, reaching 33%. There was no statistically significant agreement between anti-N seropositivity and reported COVID-19. Higher anti-spike concentrations and lower COVID-19 incidence was seen in the older vaccinees. It was noted that only subjects boosted between days 360 and 720 showed an increase in anti-spike IgG concentrations. The higher antibody concentrations (median 7440 BAU/mL) on day 360 were noted in participants not infected over the following year. Vaccination, including booster administrations, and natural, even unrecognized, contact with SARS-CoV-2 entwined two years after the primary vaccination, leading to high anti-spike antibody concentrations.

## 1. Introduction

Since the beginning of the coronavirus disease 2019 (COVID-19) pandemic in March 2020, by February 2024, more than 7 million people died from this disease. The dynamics of the severe acute respiratory syndrome coronavirus 2 (SARS-CoV-2) spread, and the toll this virus took, were substantially reduced by the introduction of widespread vaccinations [1] at the beginning of 2021.

The vaccinations proved effective [2,3,4]. In Poland, similarly to other European countries, after the peaking COVID-19 cases and deaths between the end of 2020 and the beginning of 2021, a substantial drop in the numbers in late spring–summer of 2021 was observed due to vaccinations [2]. The booster dosing recommendation was announced by the European Medicines Agency and additional, optional shots were offered in Poland at the beginning of November 2021. The following months brought new variants and subvariants, but the death toll never reached the pre-vaccination levels, which may be attributed to both diminished viral virulence and the population immunity [5,6,7,8], building upon the foundation of vaccination, further booster dosing, and immunity acquired through natural contact with SARS-CoV-2 [9,10].

Over the history of the pandemic, a prominent space in laboratory medicine was occupied by serological testing. The detection of specific SARS-CoV-2 antibodies proved useful in retrospective COVID-19 diagnosis [11,12], epidemiological studies [13], and the tentative assessment of vaccination-conveyed protection against COVID-19 [14,15].

At the beginning of the pandemic, laboratory diagnosis of COVID-19 was based on PCR testing, but the scarcity of testing sites led to the development of auxiliary means of diagnosis—lateral flow chromatography assays (LFIAs). These allowed for a qualitative assessment of IgG and IgM antibodies of poorly defined specificity. The LFIAs proved useful in their initial role [16,17], but they were shortly replaced by automated immunoassays [18,19], specific for either spike- or nucleocapsid-recognizing antibodies. After the breaking point, which was the appearance of spike-protein-based mRNA COVID-19 vaccines, the antibodies started to serve as tests assessing the immunogenicity of the vaccinations and potentially as indicators of COVID-19 protection. It was recommended to use anti-spike, WHO International Standard (IS)-standardized assays, allowing for a quantitative measurement of IgG-class antibodies [20,21]. Multiple papers have been published assessing the humoral response to COVID-19 vaccination [22,23,24,25], especially at the peak of the immune response after the primary vaccination and then following booster shot administrations [26,27,28].

Interestingly, the widespread vaccinations with mRNA vaccines led to the resurrection of anti-nucleocapsid (N) assays. In vaccinated, anti-spike IgG-positive individuals, the presence of anti-N antibodies allows for a retrospective COVID-19 diagnosis or SARS-CoV-2 contact tracing [29].

In comparison to anti-spike antibodies, anti-nucleocapsid antibodies have been reported to show a higher degree of cross-reactivity with other coronaviruses; their levels in the blood wane quicker after infection [30] and depend on the severity of the disease [31]. Anti-spike antibodies have been considered crucial for protection, as they prevent SARS-CoV-2 entry into the host’s cells, and their concentration has been correlated with sera neutralization capability [32,33]. Therefore, research has been focused on anti-spike antibodies, whereas anti-nucleocapsid immunoglobulins tend to be used solely as indicators of natural contact with SARS-CoV-2.

This study follows a cohort of healthcare workers vaccinated against COVID-19 at the beginning of 2021 [14]. The anti-SARS-CoV-2 antibodies directed at spike and nucleocapsid antigens have been monitored since with commercially available immunoassays. This stage of the study—two years after the primary vaccination—focuses on the relationship between anti-S and anti-N antibodies and booster dose acceptance, as well as breakthrough SARS-CoV-2 infections. 

## 2. Materials and Methods

### 2.1. Participants and Study Design

The study participants (*n* = 99) were recruited from healthcare workers fully vaccinated against COVID-19 at the beginning of 2021 with the Pfizer-BioNTech Comirnaty vaccine, with two doses administered 21 days apart. This cohort was enrolled over the period of January–March 2021 and has been continuously followed since, with anti-SARS-CoV-2 antibody testing performed on day 0 (day of the first vaccine dose administration), at the peak of the response to the full vaccination (day 30), and then at the consecutive timepoints: days 240, 360, and 720 after the first dose administration.

The mean age of the study group two years after the primary vaccination was 46 years (25–76); 84 subjects were under 60 years old and 15 over 60 years old. The cohort consisted of 85 females and 14 males. 

The study participants filled out questionnaires on their history of COVID-19 booster vaccinations, as well as SARS-CoV-2 and other respiratory tract infections. Out of the 99 participants, 66 reported accepting one booster dose of a COVID-19 vaccine, and 24 reported 2 booster shots. Nine vaccinees were not boosted over the course of this study.

The boosters became available after day 240 of this study, and 82 out of 99 participants accepted the third vaccine dose within four months (between days 240 and 360). A further 32 boosters were accepted between days 360 and 720.

Analysis of the questionnaires indicated that thirty-nine individuals had never suffered from COVID-19. Fifty subjects reported having COVID-19 once, including twelve people before the first vaccination. There were nine individuals who had had COVID-19 twice and one person had had it three times.

All subjects provided informed consent to their participation in this study. Ethical approval of this study was obtained from the Bioethics Committee of Andrzej Frycz Modrzewski Krakow University, Krakow, Poland.

### 2.2. Laboratory Testing

The antibody testing was performed with two commercially available immunoassays: the LIAISON^®^ SARS-CoV-2 TrimericS IgG (manufactured by DiaSorin S.p.A, Saluggia, Italy) and the SARS-CoV-2 IgG (manufactured by Abbott, Sligo, Ireland). The TrimericS IgG assay measures the concentration of the anti-spike (S) SARS-CoV-2 IgG antibodies. This type of antibody is produced in response to COVID-19 vaccination, as well as after SARS-CoV-2 infection. The Abbott assay detects the presence of the anti-nucleocapsid (N) SARS-CoV-2 IgG antibodies, which are induced after SARS-CoV-2 contact, but not after mRNA vaccine inoculation.

The testing with SARS-CoV-2 TrimericS IgG was performed on the day of blood collection. The remaining serum samples were aliquoted and frozen (−20 °C) and then tested with the Abbott assay. Laboratory processes were performed according to the manufacturers’ instructions. 

The LIAISON^®^ SARS-CoV-2 TrimericS IgG assay was run on the LIAISON^®^ XL analyzer (DiaSorin S.p.A, Saluggia, Italy). This chemiluminescent immunoassay quantitatively measures IgG-class antibodies recognizing trimeric spike (S) proteins. The measured signal is proportional to the concentration of the antibodies in the sample, and the results are expressed in Binding Antibody Units (BAU/mL). Results higher than 33.8 BAU/mL are defined as positive. Results higher than 520 BAU/mL were correlated by the manufacturer with the microneutralization titer of 1:80. The assay’s quantification range is between 4.81 and 2080 BAU/mL, and samples exceeding the upper quantification limit (UQL) were retested after dilution at 1:20, according to the manufacturer’s instructions. 

The Abbott chemiluminescent microparticle immunoassay (CMIA) detects IgG antibodies against the SARS-CoV-2 nucleocapsid. The results are expressed as indices, calculated as a ratio of sample and calibrator signals. The cut-off for positive result is 1.4. The testing was run on an Abbott Architect i2000sr analyzer (Abbott, Sligo, Ireland).

### 2.3. Statistical Analysis

A Wilcoxon test was used to verify the statistical significance of the differences in anti-S antibody concentrations between days 720 and 30, and 720 and 360, as well as in anti-N antibody titers between days 360 and 720. A U Mann–Whitney (UMW) test and Kruskal–Wallis (KW) test were used to verify the statistical significance of the differences in anti-spike SARS-CoV-2 IgG antibody concentrations between the subgroups.

Correlation between variables was assessed with Spearman’s coefficient of rank correlation. 

A Chi-squaretest was used to verify the statistical significance of the agreement between anti-N seropositivity and reported COVID-19 on day 720. A maximum-likelihood Chi-squaretest was used to verify the statistical significance of the relationship between the age group and the number of vaccine doses received, and also to compare the COVID-19 frequency between the age groups.

The significance level was set to 0.05. The statistical analyses were performed with STATISTICA software ver. 13 (TIBCO Software Inc., Palo Alto, CA, USA).

## 3. Results

### 3.1. Anti-Spike and Anti-Nucleocapsid SARS-CoV-2 IgG Concentrations Two Years after the Primary Vaccination

Two years after the first vaccine dose administration, 100% of the subjects were positive for anti-spike SARS-CoV-2 IgG and the antibody level was still high; the lowest concentration noted was 99 BAU/mL, and this was the only result below 520 BAU/mL (value correlated by the producer with a high neutralization capability in the microneutralization assay). The highest observed concentration was 32,400 BAU/mL. The median anti-S SARS-CoV-2 level on day 720 (3600 BAU/mL) was comparable (Wilcoxon test, *p* = 0.4906) to the peak concentration seen after the full primary vaccination on day 30 (3500 BAU/mL), as well as to the level observed a year before, on day 360 (4070 BAU/mL), after the booster shots had become widely used (Wilcoxon test, *p* = 0.4385). Between days 240 and 720, 114 booster doses were accepted by the study participants (82 in the first 120 days) and only 32 booster doses were given during the last 360 days, which was not enough to cause a further increase in the anti-S concentration.

**Figure 1:**
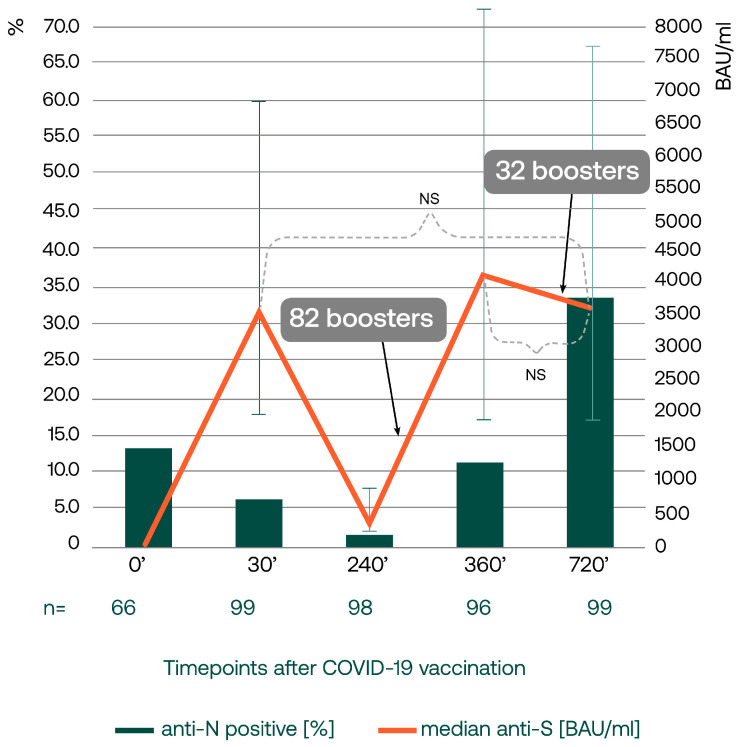
The concentrations of anti-spike SARS-CoV-2 IgG antibodies (orange line) at different timepoints after the first COVID-19 vaccination are presented as medians and interquartile ranges (q25, q75). Over the course of the study, peaking anti-S concentrations are seen after the second dose (day 30) and then after the booster shots became available. The differences between the median anti-S antibody concentrations were not statistically different between days 30, 360, and 720. Altogether, by day 720, 90 subjects were boosted once or twice. Thirty-two boosters accepted between days 360 and 720 were delivered as the fourth doses to 22 vaccinees and as third doses to 6 subjects, and two participants received their third and fourth doses between days 360 and 720. The percentage of the subjects testing positive for anti-nucleocapsid SARS-CoV-2 IgG antibodies (green bars) increased visibly over the last year of the observation, although this change was not statistically significant. However, the median anti-nucleocapsid IgG titer increased from 0.02 by day 360 to 0.65 by day 720 (Wilcoxon test, *p* = 0.00001).

Simultaneously to a slight drop in anti-S concentration between days 360 and 720, a substantial increase in the seropositivity of anti-nucleocapsid SARS-CoV-2 IgG was noted. The percentage of individuals with anti-N titers over 1.4 (threshold for positive result) two years after vaccination was the highest of all timepoints of the study (33%), pointing toward many new SARS-CoV-2 infections (Figure 1).

### 3.2. Relationship between Anti-Nucleocapsid SARS-CoV-2 IgG and the Reported COVID-19 Incidence

In addition to the increasing anti-N seropositivity, the reported incidence of COVID-19 over the last year of observation also grew. As many as 39 subjects declared testing-confirmed COVID-19 between days 360 and 720. 

We assessed the agreement between anti-N seropositivity on day 720 and the reported COVID-19—confirmed by Ag or PCR testing—over the last year. Anti-nucleocapsid antibodies were detected in 21 individuals who did not report COVID-19, indicating many undiagnosed COVID cases. On the other hand, out of 39 subjects who reported COVID-19 between days 360 and 720 (recent convalescents), only 11 were seropositive for anti-nucleocapsid IgG on day 720. There was no statistically significant agreement between anti-N seropositivity and reported COVID-19 (Chi-square test, *p* = 0.5918) on day 720. However, in 24 out of 26 COVID-19-reporting but anti-N-negative individuals, an increase in anti-N titers was noted between days 360 and 720, which might indicate their seroconversion and then seroreversion over this period. However, there was no correlation between anti-nucleocapsid antibody titers and the number of days passing since COVID-19 diagnosis (Spearman rank correlation, r = −0.08, *p* = 0.636) [Supplementary Figure S1]. 

### 3.3. Factors Influencing Anti-Spike SARS-CoV-2 IgG Two Years after Vaccination

We analyzed factors potentially affecting the anti-spike antibody titer 2 years after the vaccination. The median anti-S SARS-CoV-2 IgG concentration on day 720 in subjects who reported contracting COVID-19 (3140 BAU/mL) over the last year was not statistically different from that observed in the participants apparently not infected (3850 BAU/mL) and did not vary between participants seropositive and seronegative for anti-nucleocapsid antibodies. We found no significant correlation between anti-S and anti-N titers on day 720 either (Spearman rank correlation, r = 1.1254, *p* = 0.2158) [Supplementary Figure S1]. The anti-spike IgG concentration did not depend on the sex of the subjects, the number of vaccine doses received and the time when the last booster dose was accepted, or a recent respiratory tract infection history (Table 1).

Interestingly, the anti-S concentrations on day 720 decreased with the number of COVID-19 diagnoses over the whole course of the study; subjects who suffered from COVID-19 two or more times had the lowest anti-spike concentrations, although this finding did not reach statistical significance (Kruskal–Wallis test, *p* = 0.0642) (Table 1).

#### 3.3.1. Anti-S SARS-CoV-2 IgG in Subgroups with Different COVID-19 Incidence

**Figure 2:**
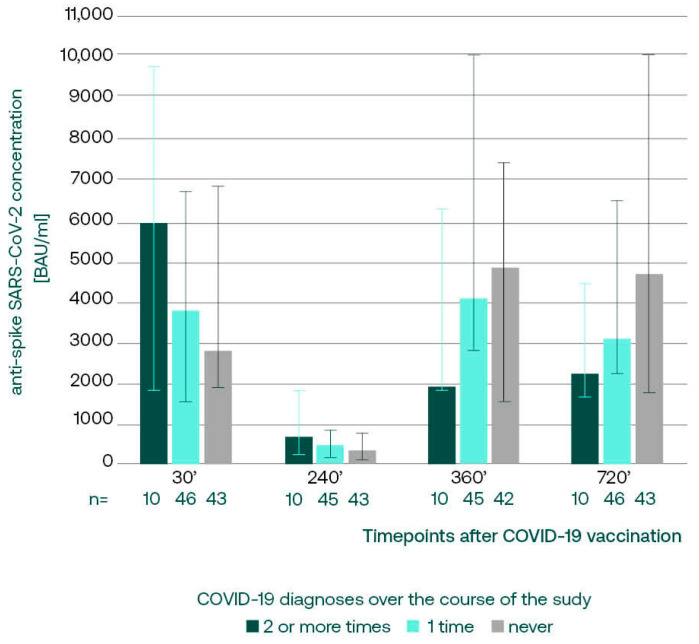
The median (q25, q75) anti-S antibody concentrations assessed retrospectively, in subgroups divided on the basis of the overall COVID-19 incidence reported on day 720.

We retrospectively analyzed the anti-S antibody concentrations in subgroups divided on the basis of the overall COVID-19 incidence over the course of the study. We noticed that multiple convalescents, presenting with the lowest titers on day 720, had peaking concentrations at the beginning of the study (Figure 2). This might have been related to the fact that 4 out of 10 participants suffered from COVID-19 prior to vaccination, in comparison to 24% in the one-time convalescent subgroup. The subsequent immunizations in this subgroup led to less pronounced increases in anti-spike IgGs, regardless of the fact that 9 out of 10 subjects reported COVID-19 between 360 and 720, and only 1 subject did not accept booster vaccinations in this period.

#### 3.3.2. Anti-S SARS-CoV-2 IgG, Booster Acceptance, and COVID-19 Incidence in Age Subgroups

Out of the analyzed epidemiological factors, only age of the subjects was demonstrated to significantly influence the level of anti-S antibodies on day 720. Participants older than 60 y.o. showed much higher antibody concentrations in comparison to the individuals below 60 y.o. (U Mann–Whitney test, *p* = 0.0010) (Table 1). There was also a statistically significant correlation (Spearman rank correlation, r = 0.3457; *p* = 0.00045) between the anti-S SARS-CoV-2 concentrations and the age of the subjects.

**Figure 3:**
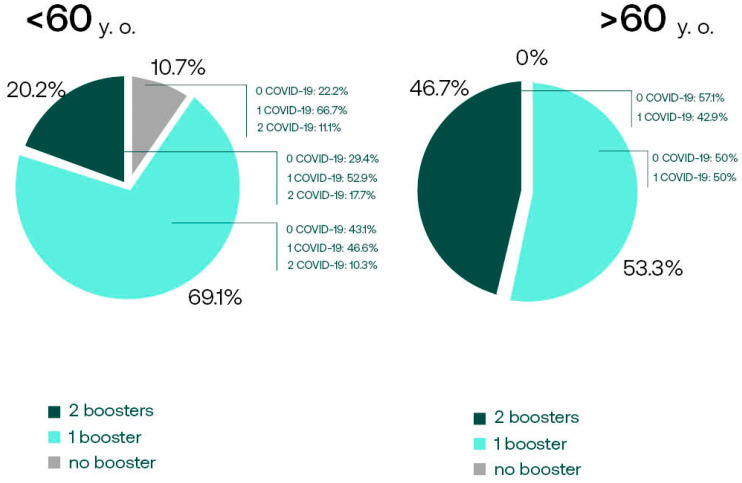
The comparison of the booster acceptance in the subgroups of younger (*n* = 84) and older (*n* = 15) subjects. COVID-19 frequency in subgroups is also shown.

We analyzed the booster acceptance rate between the age subgroups. Out of 15 subjects 60 y.o. or older, 7 received four vaccine doses (46.7%), whereas the second booster shot was accepted by only 20% of the younger vaccinees (Figure 3). The relationship between the age group and the number of vaccine doses received was statistically significant (maximum-likelihood Chi-squaretest *p* = 0.04433). 

Interestingly, the incidence of COVID-19 in the younger participants was higher (61.18% vs. 53.3%), and only the younger subjects had recurrent infections (10 out of 85 had COVID-19 two or three times). However, there was no statistically significant relationship between COVID-19 frequency and the age group (maximum-likelihood Chi-squaretest, *p* = 0.17033).

### 3.4. Anti-S SARS-CoV-2 Changes between Days 360 and 720, in Relation to Booster and Convalescence Status

**Figure 4:**
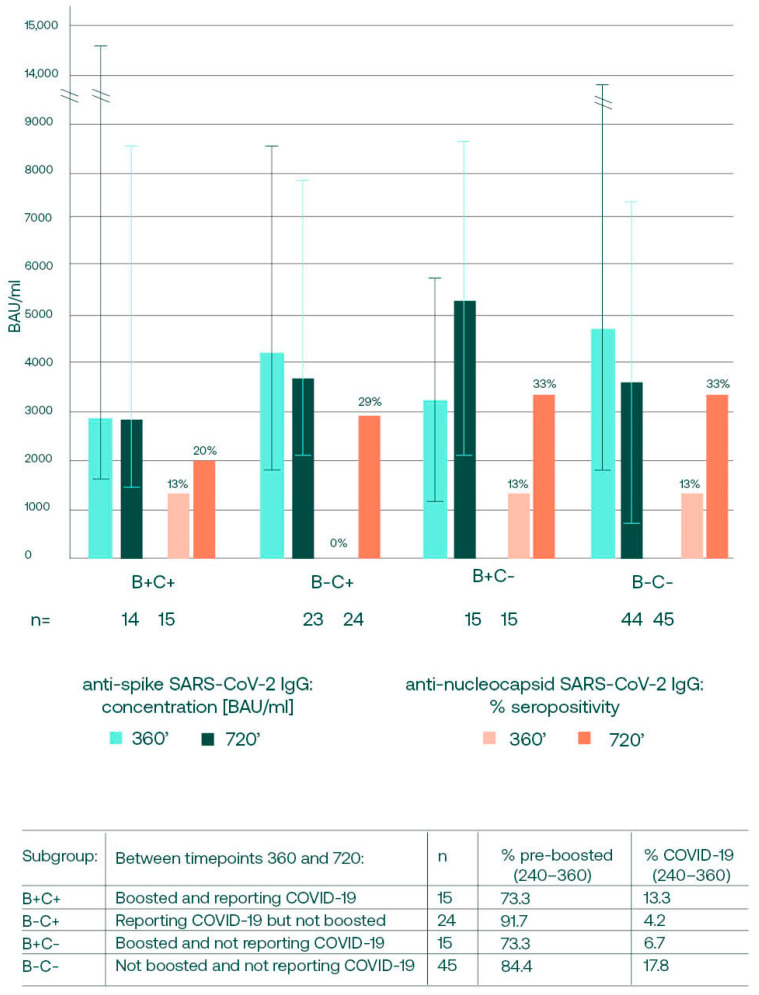
The comparison of anti-spike antibody median (q25, q75) concentrations and the percentages of anti-N seropositivity between days 360 and 720 in 4 subgroups. In all of the subgroups, the anti-N seropositivity grew over the last year of observation, whereas an increase in anti-S median concentrations was only noted for the boosted subgroups. The differences between the timepoints were not statistically significant. Subgroups with the higher booster acceptance rate prior to day 360 (“pre-boosted” 240–360), had higher anti-spike median concentrations on day 360. However, the higher pre-boosting percentages were seen in the subgroups who did not decide to accept additional shots in the following year, which led to the lower concentrations of anti-spike antibodies on day 720.

We also aimed to assess whether the direction (rise or drop) and the magnitude of anti-spike antibody concentration changes between days 360 and 720 were affected by the boosting and COVID-19 convalescence in this period. The subjects were divided into subgroups, as shown in Figure 4’s legend, and the anti-S and anti-N antibody changes over the last year of observation were analyzed.

The antibody changes between days 360 and 720 were statistically insignificant for all of the subgroups. We noticed that an increase in anti-S IgG concentration over the last year was only noted in the boosted subgroups. In the subgroups not receiving booster shots, decreases were noted, even in individuals who reported COVID-19 between days 360 and 720. Interestingly, when the anti-nucleocapsid SARS-CoV-2 IgGs were analyzed, it was noticed that the percentages of individuals seropositive for anti-N grew in all of the subgroups. This natural contact with the virus might have induced anti-spike antibody production, resulting in a high concentration on day 720, even in the subgroup not boosted and not reporting COVID-19. 

### 3.5. Anti-S SARS-CoV-2 IgG Concentrations on Day 360 and the Future Infections

Since the main reason for the interest in anti-spike SARS-CoV-2 antibodies is their role in COVID-19 protection, we analyzed whether there is a relationship between the anti-S antibody concentrations on day 360 and SARS-CoV-2 infections over the following year. We found statistically insignificant (U Mann–Whitney test, *p* = 0.0872) higher anti-spike SARS-CoV-2 median concentrations on day 360 in patients not infected with SARS-CoV-2 over the following year (median 7440 BAU/mL) in comparison to the group who became infected (median 3780 BAU/mL). 

Out of 39 subjects who reported COVID-19 between days 360 and 720, only 4 suffered severe disease and 35 described their symptoms as mild. The concentration of anti-S SARS-CoV-2 antibodies on day 360 was lower in the severe case group (2865 BAU/mL vs. 4195 BAU/mL). This finding was not statistically significant (U Mann–Whitney test; *p* = 0.6422).

## 4. Discussion

There is a plethora of research papers describing the humoral response to COVID-19 [11,12] and COVID-19 vaccination [9,34]. Our data show that these two factors—vaccination, including booster administrations, and natural, even unrecognized, contact with the SARS-CoV-2 virus—entwine two years after the primary vaccination and lead to high anti-spike antibody concentrations.

On day 720, the majority of the study participants had been boosted at least once and there were only 9 (out of 99) subjects who had not received any booster shots of the COVID-19 vaccine. However, the level of anti-spike (S) SARS-CoV-2 antibodies in this group did not differ significantly from that observed in the individuals boosted one or two times. We considered that apart from the sole fact of being boosted, the timing of the booster could have affected the antibody concentration observed on day 720, as it has been reported that the effect of the additional dose wanes within a few months [35,36]. Surprisingly, the median concentrations in non-boosted participants, those boosted more than one year prior to the testing, and those boosted within the last year were almost identical.

As the antibody concentration in the non-boosted subjects was still high, and the substantial increase in the seropositivity for anti-nucleocapsid (N) SARS-CoV-2 IgG was seen in our cohort, we speculated that the spread of the virus contributed to the median anti-spike antibody concentrations observed on day 720, especially in the non-boosted individuals.

The increasing seropositivity in anti-N antibodies was seen over the last year of observation independently of the confirmed convalescence or the booster status (Figure 4) in this period. The natural contact with the virus in mRNA vaccine recipients may be detected as a presence of anti-nucleocapsid SARS-CoV-2 IgG [29,37], and it has been shown that the level of anti-nucleocapsid SARS-CoV-2 antibodies correlates positively with the concentration of anti-spike IgG [38]. The last year of our observation fell into the period with a predominance of highly contagious yet less virulent SARS-CoV-2 variants [39] [Supplementary Figure S2], and in addition to the increased seropositivity, a rise in median anti-N titers was observed. This might mean that in the areas of implemented booster dosing, the wide spread of the virus variants caused asymptomatic infections. This stimulates SARS-CoV-2 immunity and manifests as high anti-spike antibody concentrations, even in subjects not recently boosted. However, when the changes in anti-S concentrations between days 360 and 720 were analyzed separately for four subgroups divided based on recent (12 months) boosting or convalescence, it was noted that only the participants receiving booster doses showed an increase in anti-S concentrations, what indicates that the boosters are still necessary to keep high antibody concentrations. Boosting not only leads to higher binding anti-S concentrations, but also increases the neutralization properties of antibodies, providing a higher degree of protection from symptomatic infection with SARS-CoV-2 variants [40,41].

Natural SARS-CoV-2 infection stimulates the production of anti-spike antibodies. Our observations from the beginning of this study [14], as well as papers published by other research groups [26,34,42], demonstrated that in COVID-19 convalescents, the immune reaction to the first vaccine dose was more pronounced than in vaccine-naïve recipients, and the second dose did not cause a substantial increase in IgG levels. Similarly, the analysis of antibody concentrations on day 720 revealed that the more times a participant had suffered COVID-19, the lower the anti-spike concentration was on day 720. This is an interesting phenomenon, possibly attributed to the immune system adjusting to the frequent contact with the virus and orchestrating its function through different mechanisms, not necessarily increased binding anti-spike antibody production [43]. On the other hand, the lower increases in the concentration of anti-spike antibodies in this subgroup could have been the reason for the higher frequency of COVID-19.

Apart from natural or vaccination-related contact with the virus, factors such as overall health, sex, and age influence the humoral response of an individual. Since our cohort consisted of apparently healthy healthcare professionals, we analyzed only the influence of sex and age. 

When measured at the peak of the vaccination response, the anti-spike SARS-CoV-2 concentrations are generally reported to be higher in younger individuals, and in women in comparison to men [44,45,46,47,48]. In our cohort, the relationship between anti-S IgG concentrations and sex was equivocal. Previously, we reported [14] that females tended to have statistically higher antibody titers up to 4 months post primary COVID-19 vaccination. Over the two-year course of this study, the relationship between anti-S IgG concentration and sex lost significance [44,45].

In age subgroups, we observed statistically significantly higher antibody titers in the younger (below 60 y.o.) vaccinees, but only at the peak of the primary response to the vaccination [14]. Over the following months, the antibody titers in the age subgroups became comparable by day 360, and by day 720, the older vaccinees surprisingly demonstrated significantly higher anti-S SARS-CoV-2 antibody concentrations. This could have been caused by the higher rates of booster dose acceptance in this age group. Importantly, the higher antibody concentrations in subjects over 60 y.o. in our study were accompanied by a lower COVID-19 incidence, possibly also stemming from a higher compliance with prophylaxis measures in this subgroup. This finding is in line with that reported by Staerke et al. on the association between increasing age and reduced risk of breakthrough infections [49]. Hence, it might be suspected that age influences the antibody levels indirectly, although with different behavioral patterns, stimulated by the elderly-dedicated prophylaxis campaigns released by both international and governmental agencies.

In addition to analyzing the influence of different factors on anti-spike antibody concentrations, we attempted to investigate whether high anti-spike antibody titers prevented SARS-CoV-2 infection in our cohort. It has been shown previously [50,51] that the risk of symptomatic COVID-19 disease decreases with increasing levels of anti-S IgG. It has also been reported [52,53] that a low level of neutralizing and anti-spike IgG antibodies may indicate a risk of breakthrough infections. Stærke et al. showed that this was true for the Delta variant of SARS-CoV-2, but the risk of breakthrough infection with Omicron was not related to anti-S IgG concentrations [49]. Although some efforts have been put towards establishing an antibody protective titer [51,54,55,56], neither a universal cut-off for protection nor a definition of a “high” antibody concentration have been proposed yet. Dimeglio et al. reported that anti-S-RBD antibody concentrations of 1700 BAU/mL and above provided full protection in subjects followed-up for a median of 275 days (ca. 9 months) [55]. For immunoassays similar to those used in this study (LIAISON^®^ SARS-CoV-2 TrimericS IgG), the concentration of 899 BAU/mL has been suggested to confer 90% efficacy against symptomatic infection [51]. Even lower levels (520 BAU/mL) have been correlated by the immunoassay’s manufacturer with a high neutralization capability. In our study, the median anti-spike concentration measured on day 360 was 4070 BAU/mL—higher than both these values. In spite of this, between days 360 and 720, an increased rate of breakthrough SARS-CoV-2 infections was seen. However, most of the cases were asymptomatic or mild, with only four participants declaring their symptoms as severe. This observation is in line with the main aim of the vaccinations: preventing severe illness and hospitalization. Although our findings were not statistically significant, it has to be noted that the anti-S concentration was lower in subjects who became infected over the year following day 360 (3780 BAU/mL vs. 7440 BAU/mL), and it was lower in the subjects reporting severe symptoms (2865 BAU/mL vs. 4195 BAU/mL). Additionally, when the antibody titer on day 360 was compared between the subjects who became infected within 6 months, or between 6 and 12 months post measurement, we found that a lower concentration was seen in those infected sooner (3260 BAU/mL vs. 6030 BAU/mL,). Hence, our results provide some additional evidence for the relationship between anti-spike antibody concentration and COVID-19 protection and point out the importance of the “expiration date” of the antibody measurements.

Our observations indicate that immunity boosters—either in the form of additional vaccine shots or in the form of infection—are necessary to maintain protection against severe COVID-19. However, the holy grail of COVID-19 immunity correlate may be hard to grasp [57], and finding antibody titer protection from any infection has been considered unrealistic [58]. Research directed at establishing a severe-COVID-19-protective immunoglobulin titer should take into account factors associated with the virus (SARS-CoV-2 emerging variants), the vaccine (its format), and practical aspects of antibody testing, such as the accessibility of assays in real life (focusing on binding, and not neutralizing antibodies) and providing optimal timepoints for testing, allowing for the prediction of a prospective, defined immunity period. 

## Figures and Tables

**Table 1:** Median anti-spike SARS-CoV-2 IgG in different subgroups.

Subgroups		N	Median Anti-S SARS-CoV-2 IgG [BAU/mL] 2 Years after the Vaccination	Significance
Sex	Female	85	3980	NS (*p* = 0.4454) U Mann–Whitney test
	Male	14	3010	
Age	<60	84	3090	*p* = 0.001 U Mann–Whitney test
	>60	15	6460	
Number of vaccine doses received	2	9	3520	NS (*p* = 0.4175) Kruskal–Wallis test
	3	66	3140	
	4	24	4950	
Timing of the last booster	No booster	9	3520	NS (*p* = 0.5578) Kruskal–Wallis test
	Before 360	60	3660	
	After 360	30	3880	
COVID-19 history (self-reported)	No	39	4640	NS (*p* = 0.0642) Kruskal–Wallis test
	1	50	3090	
	≥2	10	2190	
COVID-19 history between days 360 and 720	Yes (self-reported)	39	3140	NS (*p* = 0.8467) U Mann–Whitney test
	No	60	3850	
Anti-nucleocapsid seropositivity on day 720	Anti-N positive	30	3010	NS (*p* = 0.8088) U Mann–Whitney test
	Anti-N negative	67	4180	
Other respiratory infections over the past 3 months	Yes	31	3140	NS (*p* = 1.0000) U Mann–Whitney test
	No	68	3850	

## Data Availability

The data presented in this study are available on reasonable request from the corresponding author.

## Supplementary Materials

The following supporting information can be downloaded at https://www.mdpi.com/article/10.3390/vaccines12050471/s1, Figure S1: Correlation analysis of the relationship between anti-nucleocapsid IgG titers on day 720 and (a) time passing since the reported COVID-19 and (b) anti-spike IgG concentrations on day 720; Figure S2: The prevalence of different SARS-CoV-2 strains in Poland over the study period.
