# Supplementary figures and images for: High Concentration of Anti-SARS-CoV-2 Antibodies 2 Years after COVID-19 Vaccination Stems Not Only from Boosters but Also from Widespread, Often Unrecognized, Contact with the Virus
Source: Vaccines (Basel). 2024 Apr 28;12(5):471. doi: 10.3390/vaccines12050471 (PMC11125768; doi:10.3390/vaccines12050471)

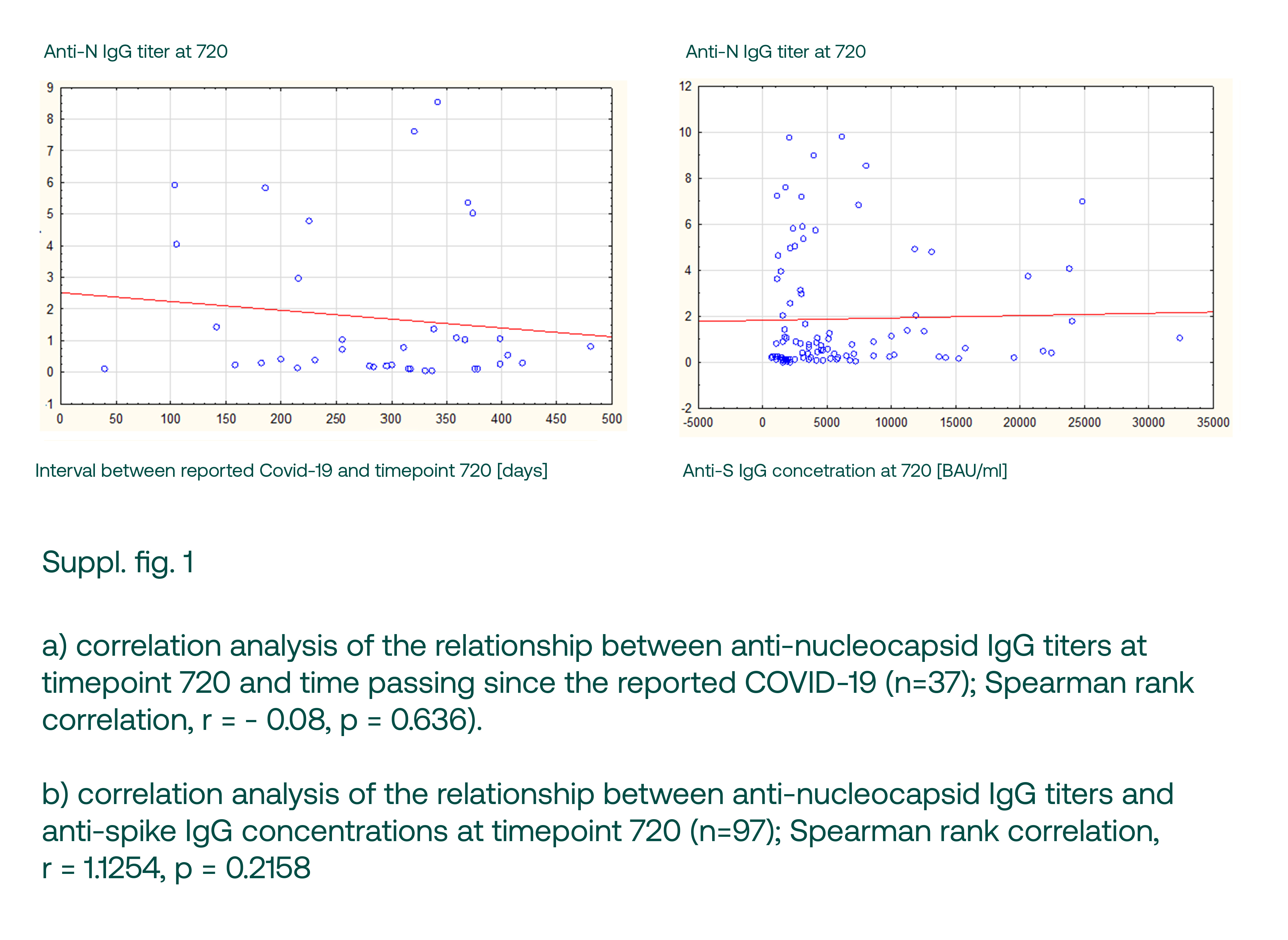

Supplement: Supplementary file 1 [file vaccines-12-00471-s001.zip › Supplementary figure S1.jpg]

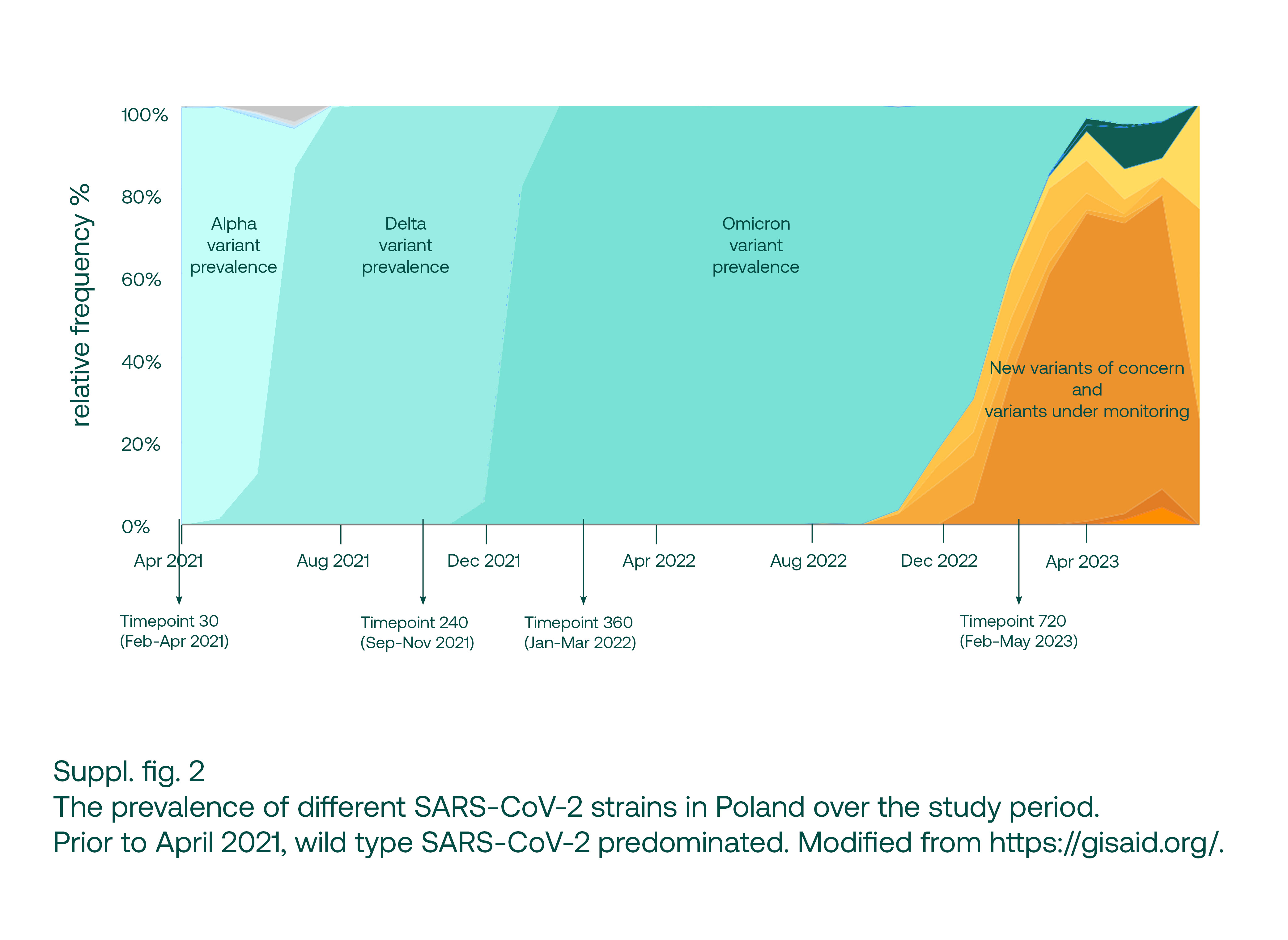

Supplement: Supplementary file 1 [file vaccines-12-00471-s001.zip › Supplementary figure S2.jpg]
